# Exploring the Links Between Biophilic and Restorative Qualities of Exterior and Interior Spaces in Leon, Guanajuato, Mexico

**DOI:** 10.3389/fpsyg.2021.717116

**Published:** 2021-08-17

**Authors:** Joel Martínez-Soto, Luis Alfonso de la Fuente Suárez, Salvador Ruiz-Correa

**Affiliations:** ^1^Department of Psychology, Universidad de Guanajuato, Leon, Mexico; ^2^Architecture School, Universidad Autónoma de Nuevo León, San Nicolás de los Garza, Mexico; ^3^Youth Innovation Laboratory, Centro Nacional de Supercómputo, Instituto Potosino de Investigación Científica y Tecnológica, San Luis Potosi, Mexico

**Keywords:** restorative environments, biophilic design, psychological restoration, built environments, green spaces

## Abstract

The interactive role of the ecological, architectural, biophilic, and sensory qualities of outdoor and indoor spaces in the restorative experiences of urban inhabitants is little known. We analyzed the restorative influence on mood states and situational stress related to exposure to vegetation proportion, spatial extension, landmark salience, biophilic architecture, people density, street visual access, olfactory pleasantness, and noise of 65 public spaces in a Mexican city. The environmental qualities of these places were analyzed with multidimensional scaling (MDS), leading to eight space categories (e.g., historic squares with biophilic architecture, large parks, street scenes, and interiors with non-biophilic architecture). Ratings of the restorative potential, mood states, situational stress, olfactory pleasantness, and noise annoyance were evaluated on such places and modeled through a structural equation modeling (SEM). The model shows that the restorative influence of the environmental qualities on moods and stress was related to a decrease in experiences of negative moods and perceived stress, and an increase of positive mood states. Based on our findings, we discuss design guidelines, emphasizing the relevance of including vegetation and built elements with biophilic qualities to create restorative environments.

## Introduction

Growing urbanization and modern lifestyles are associated with mental fatigue (White and Shah, 2019), chronic stress (Abbott, 2012), depression (Hidaka, 2012), obesity, diabetes, and hypertension (Egger, 2012). In contrast, recent studies have documented consistent dose-response positive effects of nature exposure and time experiences on indicators of physical and mental health (Hazer et al., 2018; White et al., 2019; Jones et al., 2021). Several studies have also provided additional evidence of the potential benefits of nature and nature-like infrastructure in common outdoor and indoor spaces (Bower et al., 2019; Martínez-Soto, 2019). The latter encompasses studies on psychological restoration and restorative environments, which have a long tradition of validating the psychological, physiological, and social benefits of contact with nature-like settings (Menardo et al., 2019). However, there are some limitations on capturing the diversity of restorative places and the specific qualities linked to restorative outcomes in such areas. The studies on the impact of the biophilic elements that extend the restorative effects of outdoor and indoor spaces are not fully developed (Shen et al., 2019; Marte et al., 2020). We focus on a study conducted through mobile crowdsourcing to explore the quantitative relationships between environmental variables (which include biophilic variables) and their associations with restorative features of 65 exterior and interior spaces in a city located in central Mexico.

### Psychological Restoration and Restorative Environments

Psychological restoration is the renewal of individual depleted resources caused by adaptation of ordinary demands, such as stressors and challenging tasks (Honold et al., 2016). Restoration involves short-term, mood-like outcomes related to physiological, affective, and cognitive states (Kaplan and Kaplan, 1989). A restorative environment is one whose qualities promote psychological restoration (Hartig, 2011).

The psychophysiological stress recovery theory (PSRT) and Kaplan's attention restoration theory (ART) are the two dominant theories on restorative environments. Based on the biophilia hypothesis, the PSRT suggests that human beings have a predisposition for unthreatening natural settings because an innate preference for natural environments is an advantage for early humans (Ulrich, 1983). The theory argues that exposure to nature can automatically reduce stress of people (Ulrich, 1983). Nature post-stress recovery includes psychological, affective, and cognitive changes related to the recovery from excessive physiological arousal, reduced negative emotions, and an increase of positive feelings (Ulrich et al., 1991). Psychophysiological stress recovery theory relies on successful studies where active or passive transactions with natural settings show affective results measured through self-reported mood scales (Huang et al., 2020). The ART conceptualizes restoration as a process that returns cognitively fatigued people or a person with a high negative affect to a mental state wherein these affectations are reduced (Rydstedt and Johnsen, 2019). This theory suggests that exposure to an environment with restorative attributes helps restore directed attention fatigue. Qualities linked with experiences of being away, fascination, compatibility, and extent are essential to restoration (Kaplan and Kaplan, 1989). These components quantifying the restorative potential are mediators between the physical environmental attributes and restorative outcomes (e.g., Hartig et al., 1997; Lindal and Hartig, 2013; Martínez-Soto et al., 2014b; Marselle et al., 2016). Recent approaches to evaluate the restorative capacity of urban environments have explored various experimental settings at acute and long cumulative mental fatigue or stress levels, considering brief or repeated transactions with restorative periods (Pasanen et al., 2018a; Hunter et al., 2019). These include contrasting nature vs. stressful and controlled environments (Pasanen et al., 2018a). Evaluation of the restorative capacity of urban environments has been conducted in need for restoration conditions (Jiang et al., 2014; Stigsdotter et al., 2017; Subiza-Pérez et al., 2021) and without the need for restoration (Cheon et al., 2019; Kang and Kim, 2019). The need for restoration is commonly induced by causing a psychological deficit of acute mental fatigue or psychological stress. The studies concluded that exposure to restorative built environments relates to psychophysiological changes associated with the restorative potential of a place. Namely, psychophysiological outcomes are related to an improved sense of relaxation and revitalization (Johansson et al., 2011; Van den Berg et al., 2014), a decrease in negative and clinically relevant mood states, reduction in perceived stress (Brooks et al., 2017; Hazer et al., 2018; Bielinis et al., 2021), and an increase in attentional performance (Subiza-Pérez et al., 2021).

Research trends in restorative environments group into four categories: (a) comparative studies on advantages of restoration of natural vs. urban built settings (Ulrich et al., 1991; Pasanen et al., 2018a), (b) studies on the exposure to multiple green space typologies (Matsuoka and Kaplan, 2008; Honold et al., 2016), (c) studies relating to the dichotomy nature vs. architectonic/historic built environments (Karmanov and Hamel, 2008; Scopelliti et al., 2019), and (d) studies in which architectural qualities of indoor and outdoor scenarios result in restorativeness beyond greenness (Lindal and Hartig, 2013; Subiza-Pérez et al., 2021).

### Restorative Built Spaces Features

Restorative built spaces include green spaces (Lorenzo et al., 2016; Stigsdotter et al., 2017), residential and non-residential streetscapes (Gidlow et al., 2016; Kabisch et al., 2021), blue spaces (Gidlow et al., 2016; Subiza-Pérez et al., 2020; Kajosaari and Pasanen, 2021), cultural and historic places (Ouellette et al., 2005; Clow and Fredhoi, 2006; Hidalgo et al., 2006; Fornara and Troffa, 2009; Herzog et al., 2010; Xu et al., 2018; Subiza-Pérez et al., 2021), and, in a lesser extent, indoor built spaces (e.g., shopping malls and coffee shops, Staats et al., 2016; Payne and Guastavino, 2018; work environments, Raanaas et al., 2011; Korpela et al., 2014). Restorative attributes of these space categories relate to their aesthetic qualities (Galindo and Hidalgo, 2005; Lindal and Hartig, 2013; Subiza-Pérez et al., 2020), the degree of naturalness, diversity of vegetation, tree cover density, functionality, and extension (Van den Berg et al., 2014; Ettema, 2016; Lorenzo et al., 2016). These attributes have been consistently studied as predictors of restorative experiences. However, other environmental qualities beyond nature traits significantly contribute to the restorative potential of a place. The levels of exteriority and visibility, which promote visual and locomotive permeability, relate to the perceived restorativeness (Hauru et al., 2012), the restoration likelihood (Lindal and Hartig, 2013), environmental preferences (Herzog, 1992), the attentional performance, and mood states (Huang et al., 2020). Psycho-environmental indexes of the enclosure are used to quantify these attributes (Subiza-Pérez et al., 2021). Walkability potentializes restorative effects (Gatersleben and Andrews, 2013; Zuniga-Teran et al., 2017; Lin et al., 2019) and is a predictor of better health (Honold et al., 2016). The presence of landmarks in urban spaces (see Bala, 2016) facilitates wayfinding, promoting restorative outcomes (Gatersleben and Andrews, 2013; Pasanen et al., 2018a). Human-made buildings that sustain various social and historic meanings could reach similar restorative potential as natural areas (Xu et al., 2018). Psychological or physiological impairment has not been observed in these spaces (Stigsdotter et al., 2017). Street proximity relates to deleterious restoration effects (Bornioli et al., 2018). People density also hinders restorative environmental outcomes (Staats and Hartig, 2004; Galindo and Hidalgo, 2005).

Non-visual modalities, such as the sense of hearing and smell, play an essential role in restorative experiences. Natural sounds have a restorative potential (Ratcliffe et al., 2016) due to induced mood and cognitive recovery effects (Benfield et al., 2014; Abbott et al., 2016; Shu and Ma, 2018). Odors directly influence the experiences of people of their surrounding environment and proportionate a rich set of localized information that enhances the sensory experience with the natural environment (Henshaw, 2014; Shaw et al., 2015). Olfactory pleasantness from natural smells enhances positive moods and reduces negative feelings (Jo et al., 2013) and stress (Hedblom et al., 2019). Odors found in urban environments (e.g., caused by air pollution) relate to sustained stress (Hedblom et al., 2019). Therefore, olfactory sensory inputs are relevant when creating environments that reduce stress and promote restoration (Henshaw, 2014; Sowndhararajan and Kim, 2016; Truong et al., 2019).

### Biophilic Design

Wilson (1984) borrowed the term “biophilia” from Erich Fromm (1984) to suggest that human beings possess an innate tendency to seek connections with nature and other forms of life. Such tendency refers to “focus upon life and lifelike forms, and, in some instances, to affiliate with them emotionally” (Wilson, 2002, p. 134). The concept of affiliation is equivalent to a trait measure related to the experiential sense of oneness with the natural world (Wilson, 1993). These sets of experiences are used to describe the deep sensations of exploring the natural and the cognitive and affective manifestations that arise while inhabiting such environments (Appleton, 1975). From the early conceptions of biophilia to the biophilia hypothesis, how greenery in built environments affects the human connection with nature has been the main topic of interest (Söderlund and Newman, 2015). Biophilic design is an approach that attempts to connect people with nature (Kellert, 2008; Browning et al., 2014). Several elements and attributes of biophilic design have been elaborated through the intuition of the designer (for a review of such attributes, see Kellert, 2008, 2018; Browning et al., 2014; Ryan et al., 2014; Sturgeon, 2017), thus supporting the human needs of affiliation with nature through the design of indoor and outdoor built spaces (Söderlund, 2019). Some psycho-environmental measures of biophilic design are included in the biophilic quality index by Berto and Barbiero (2017). Large-scale application of biophilia can also be found in the biophilic cities movement (e.g., Beatley, 2016) and in the field of cultural ecosystem services (Chang et al., 2020). Biophilic designers incorporate biophilic qualities (e.g., the imitation and use of patterns and shapes that relate to natural elements; Söderlund and Newman, 2015) to promote similar psychological benefits to those obtained with authentic natural experiences (Salingaros, 2007). Recent work on biophilic design has investigated the psychological impact of nature-like features in built environments (Gillis and Gatersleben, 2015). These include mood and cognitive functioning improvement, increased positive emotions and creativity, and life satisfaction (Ortegón-Cortázar and Royo-Vela, 2019; Roskams and Haynes, 2020). Experimental studies have also observed low skin conductance and decreased blood pressure and heart rate (Yin et al., 2018; Shen et al., 2019). These findings indicate that an individual connection with nature could determine the ability of people to positively evaluate the restorative potential of a natural environment (Berto et al., 2018). The latter findings agree with the notion that while the biophilic affinity is focused on individual preferences linked with connectivity with nature, the restorativeness of the place is an environmental quality (Berto et al., 2018). Our research points out the role of several environmental biophilic qualities in the promotion of the cognitive, emotional, and physiological components of the recovery and restoration processes (Rydstedt and Johnsen, 2019). The latter suggests that PSRT and ART support the biophilic design practice (McSweeney et al., 2019; Shen et al., 2019; Yin et al., 2020). However, despite advances in understanding the physical and physiological effects of biophilic features in built environments, several researchers agree that further evidence is required to characterize such effects quantitatively. Existing studies have focused on measurements of the impact of biophilic qualities on the restorative responses and the overall benefits of biophilic spaces (Yin et al., 2018, 2020; Coburn et al., 2019; Shen et al., 2019).

### Study Aims and Objectives

This article identifies the biophilic design features and other environmental qualities in a sample of 65 interior and exterior spaces in a city of a middle-income country (Leon Guanajuato, Mexico). We examine the restorative influence of a set of environmental qualities that cross the barriers between the ecological, spatial, urban, architectural, psychosocial, and sensory aspects of places. A further step of this study is to establish an appropriate characterization of such scenarios according to these qualities. Along with the latter attributes, the study includes the evaluation of the places regarding their restorative quality, preference, mood states, and situational stress. A structural equation model summarizes the statistical links between the included covariates.

The research questions of this study are as follows:

Q1. How can interior and exterior spaces be categorized according to their environmental qualities?
Q2. Do the categories of place make a difference in the restorative potential, stress, and mood states as perceived by people?
Q3. How can statistical dependencies between the environmental qualities, the restorative potential, stress, and mood states be quantified through a structural equations model?
Q4. What design guidelines may be proposed on the basis of the findings of this study?

**Figure 1:**
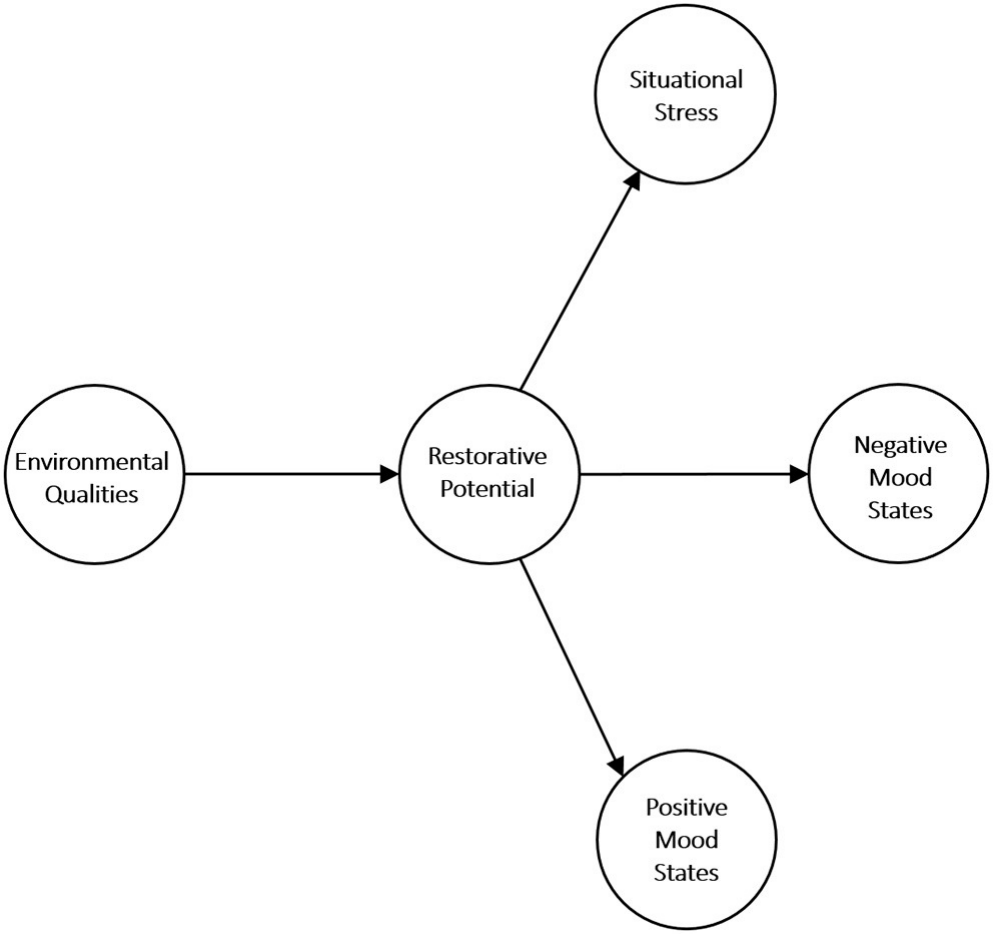
The model of the restorative influence of environmental qualities of the place on mood and stress.

As for Q3, we have proposed a conceptual model of the restorative influence of the environmental qualities of the places on mood and stress (see Figure 1). Our model departs from a social ecology theory (Stokols, 1996), which considers the categorical and dynamical inquiry about the people-environment relations based on three elements: the physical setting, the psychological process, and the psychological outcomes (Stokols, 1987; Werner et al., 2002). From a theoretical point of view, the model considers the basic assumptions of ART (Kaplan and Kaplan, 1989) and PSRT (Ulrich, 1983) to explain the restorative effects of the proposed environmental qualities on mood states and psychological stress related to the exposure with indoor and outdoor built settings. The model considers several environmental qualities, such as vegetation proportion, landmark salience, and the biophilic qualities of the built elements. Olfactory pleasantness was included as an observed dimension of the environmental qualities. The model considers the components associated with the restorative potential of a place as those that indirectly influence the relations between the environmental qualities and psychological outcomes. This consideration is supported by evidence, suggesting that the link between the environmental attributes and the restorative effects is mediated by the perception of the restorative qualities of the environment (Hartig et al., 1997; Lindal and Hartig, 2013; Martínez-Soto et al., 2014b; Marselle et al., 2016; McAllister et al., 2017; Masoudinejad and Hartig, 2020). We also hypothesized that the restorative influence of environmental qualities on moods and stress was related to decreased negative moods and perceived stress, and with higher feelings of positive mood states (McSweeney et al., 2019; Huang et al., 2020). Our proposed model implemented structural equation modeling (SEM) to evaluate how the model fits empirical data and infer the nature and characteristics of the causal associations (Figure 1).

## Methods

A total of 470 participants (out of 508 recruited volunteers) completed the whole activity by answering a survey and taking photographs of a place (262 females; *M*_age_ = 30.95, *SD*_*age*_ = 12.62; range, 16–68; 41.5% had university degree studies). Most of them were residents in the city where the study was developed (95.5%). On average, the participants had lived in the city for 20.89 years (*SD* = 16.39). Twenty-three percent of the participants were circumstantial or occasional visitors. In contrast, 77% were non-circumstantial visitors that regularly performed activities in the nearby areas of the places. Inclusion criteria comprised self-declared health and normal sensory functions. The participation was voluntary, and all the participants provided signed informed consent. The Comité Institucional de Bioética en la Investigación de la Universidad de Guanajuato (CIBIUG) provided the ethical approval for the study.

### Description of the Setting

With around 1.6 million inhabitants (Instituto Nacional de Estadística y Geografía, 2015), Leon is the most populous city in the state of Guanajuato, Mexico, and it is the seventh most populous metropolitan zone of the country (Secretaría de Desarrollo Agrario Territorial y Urbano, Consejo Nacional de Población, and Instituto Nacional de Estadística y Geografía, 2018). The town was founded in 1575 (Arias Padilla, 2020). Looking for the representativeness of the settings (Winkel et al., 2009), data were collected in 65 exterior and interior spaces in Leon. These spaces were selected to include interior and exterior places, whether contemporary or historic, with varying proportions of vegetation, different uses, and located in quite distinct areas of the city. The spaces were selected using the methodology described in Medina (2019). Most of the chosen sites are commonly used by people for leisure, shopping, and social interaction, but the study also included spaces like waiting rooms in hospitals and bus stations.

### Assessment of the Environmental Qualities of the Places

**Figure 2:**
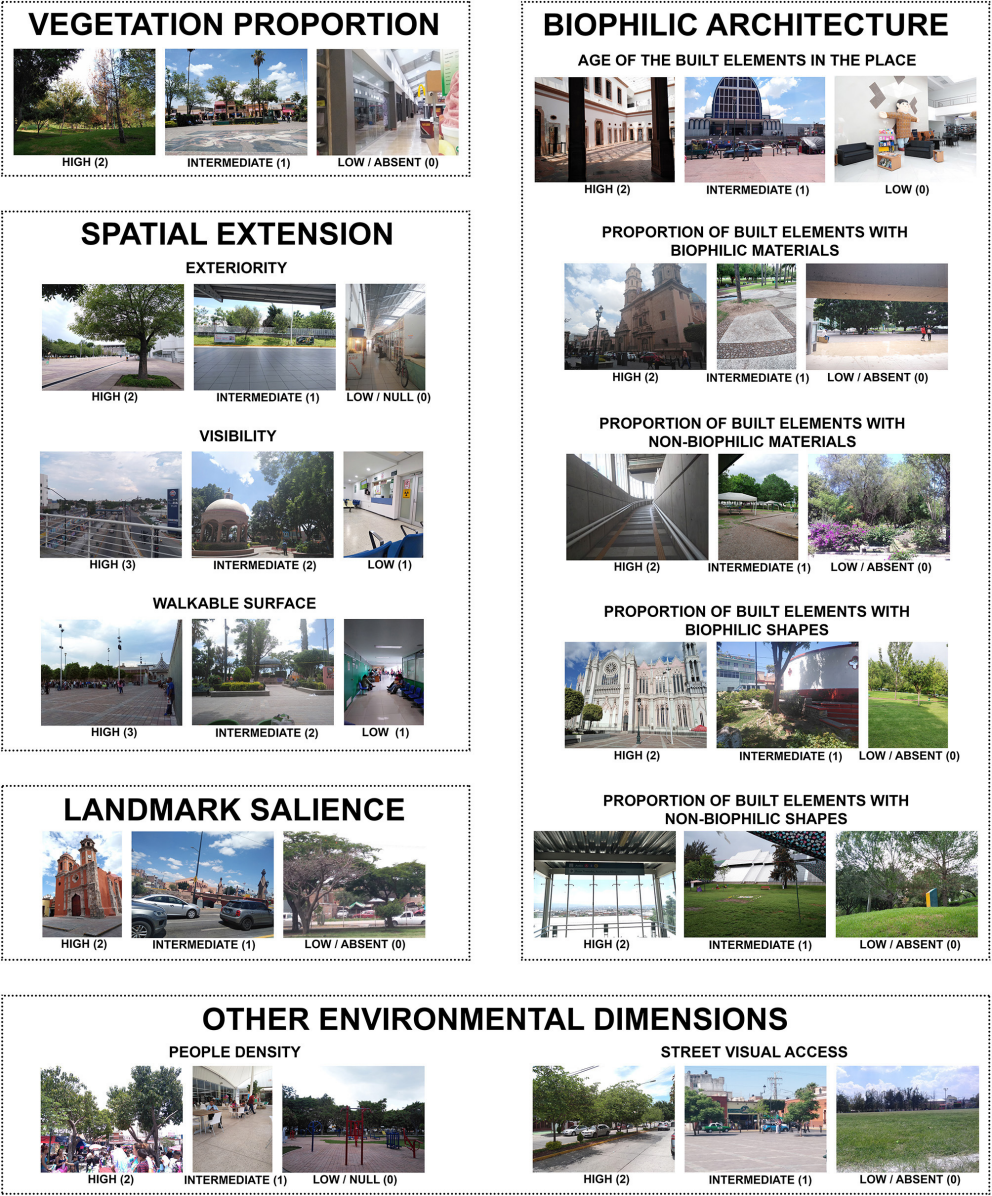
Environmental dimensions exemplified with one photograph for each of the three levels of the corresponding dimension.

The present study subjects freely visited the place, answered different psychological scales, and took photographs of their surroundings. Pictures of the spaces taken by the participant volunteers enabled a panel of expert architects and psychologists to rate a set of 12 environmental qualities (three levels for each dimension). A study in which the raters evaluated specific psycho-environmental characteristics of places *in situ* (e.g., tree density, orientation, enclosure) is San Juan et al. (2017). The environmental qualities were evaluated based on the multiple photographs of the places taken by the participants from standing or sitting points of view (Figure 2). Google Street View and aerial photographs were used when the photographs of the participants did not allow for assessing any of the 12 dimensions of a place. The selected 12 dimensions measure the physical and social qualities of places that may have restorative potential and a positive impact on the health and well-being of people. These dimensions are defined in the following paragraphs.

#### Vegetation Proportion

This dimension is the amount of greenery present in the environment relative to everything found in such an environment. This quantitative aspect of vegetation ranges from a primarily vegetation-covered environment (a high level) to a mostly built one (a low level). The intermediate level of vegetation proportion corresponds to places not surrounded by vegetation as in the high level but presenting a considerable amount of vegetation accompanying the built elements. The use of photographs to calculate vegetation proportion in places is an effective method that has been widely used. Green appearance percentages or GPs (Li et al., 2020) and urban greenery (Li et al., 2015) were measured through photographs. The latter research utilized a series of street-level images of a place obtained through Google Street View. In other studies, raters evaluated the vegetation proportion *in situ* (vegetation cover, Otero Pastor et al., 2007; density of natural elements, San Juan et al., 2017). A similar three-level evaluation of the proportion of vegetation is found in Chiang et al. (2017).

#### Exteriority

The level of contact that a place has with the open air space. A place that is not limited by walls or a ceiling would be the best example of an exterior space (a high level of exteriority). Contrarily, an environment surrounded by walls and a ceiling is considered as an interior space (a low level). The intermediate level of exteriority corresponds to the spaces possessing a ceiling but lacking walls, or the other way round. A dimension related to exteriority is that of openness: “how wide open the space in the setting appears to be” (Herzog et al., 2003, p. 161). The term exteriority was used here besides openness since the latter is related to the visibility dimension also included in this study. Meanwhile, an inversely related dimension to exteriority is enclosure. In a study about streets, Choe et al. (2020) suggest that enclosure goes from room-like to wide open. The percentage of an image covered by surfaces that block both human vision and motion (i.e., walls) is the physical attribute with the highest effect on enclosure (Stamps, 2005). Enclosure is also influenced by the proportion of sky (Ewing and Handy, 2009).

#### Visibility

This environmental dimension is the extent of the visible space limited by visual barriers that the observers encounter in a place. It is related to the isovist area (Benedikt, 1979), the quantity of space visible from a point in space. Similar to isovists, the visibility dimension considers the extension of visible space in the horizontal plane. Because the isovist area is challenging to calculate through images, the approximate length of the sight lines (the distance to the visual barriers) was used to evaluate visibility. Places with mean distances to visual barriers of <10 m correspond to the low level of visibility; those between 10 and 50 m are in the intermediate level, and places with distances of more than 50 m are in the high level of visibility. While evaluating the visibility of the places, multiple points in space were considered regarding the distances to visual barriers. The visibility of a space is evaluated separately from exteriority since an interior space may allow high visibility, as in some commercial spaces, while an exterior space like a park may have limited visibility, owing to the number of trees. Visibility relates to the dimension of visual access or visual permeability of the boundary (Stamps, 2005, 2010). A higher visual permeability relates to lower feelings of enclosure (Stamps, 2005). Herzog et al. (2003, p. 161) defined the visual access of an environment as: “how well one can see into all parts of the setting without having one's view locked.”

#### Walkable Surface

The amount of horizontal or a nearly horizontal ground plane at people's disposal for their displacement through space. Following the concept of affordances by Gibson (1968), a place with an extensive horizontal area affords unrestricted and comfortable movement in multiple directions through a vast amount of space. A place presenting an extensive ground surface without barriers to movement is included in the high level of a walkable surface. The intermediate level presents environments with moderate extensions of ground and a moderate quantity of elements restricting the movement of people. Highly restricted environments regarding the ground surface in which people find barriers to movement in close distances correspond to the low level of the walkable surface dimension. Similar concepts related to a walkable surface are unobstructed pedestrian movement (Ayataç et al., 2020), locomotive access or locomotive permeability (Stamps, 2005; Chiang et al., 2014), horizontal area (Stamps, 2010), and built surfaces for movement (Hunter and Askarinejad, 2015).

#### Landmark Salience

This dimension corresponds to the noticeability of an architectural or sculptural element observable in a place. According to Lynch (1960), a landmark is an external physical element, a point of reference in which the observer does not enter. In this study, such built elements acting as reference points may be exterior (e.g., a church, a bridge, a fountain) or interior (e.g., a stair, a distinctive wall or a roof). The perceptual salience of a built element is a required characteristic of a landmark, whether it be visual or semantic salience (Caduff and Timpf, 2008). The latter authors indicate that a landmark may contrast with the surrounding environment in its attributes or due to its spatial location. Landmark salience is related to the dimension of focality (Ulrich, 1983, p. 99): “the degree to which a scene contains a focal point, or an area that attracts the observer's attention.” Focality in scenes correlates with preference, according to Ulrich (1977). In this study, high landmark salience is possessed by places presenting a building or an element with a large scale or height, which is visible from most of the locations inside of a place. Intermediate landmark salience corresponds to places possessing a built element that is not so large in scale or is not so visible from the different locations in a place. The absence of a built element that differentiates from its surroundings corresponds to the low level of landmark salience.

#### Age of the Built Elements in the Place

The approximate time period in which such elements may be categorized, owing to their antique or contemporary style. The dimension of the age of the built elements relates to one of the biophilic design attributes described by Kellert (2008, p. 12), i.e., the historic connection to a place: “Buildings and landscapes that elicit this continuity with the past encourage the belief that the present and the future are meaningfully linked to the history of a place.” Existing studies considering the age of buildings in an environment distinguish between old and contemporary buildings (Herzog and Gale, 1996; Herzog and Shier, 2000; Ng, 2020). Nevertheless, in the present study, the age of built elements has three levels. When a place includes buildings or monuments with old styles, materials, and construction systems, it is categorized in the high level of this dimension. Old-style buildings, in most cases, are previous to the modern architecture era, which, in Mexico, began in 1925, according to Noelle (1993). The low level of the age dimension corresponds to contemporary buildings, which are of relatively recent construction of around 20 years or less. Buildings that do not possess old styles and are not of recent construction belong to the intermediate level. Most modern architecture buildings pertain to this level. These buildings often present concrete, steel, and glass, and simple shapes devoid of ornament. Coeterier (2002) found that laypeople judge the value of old buildings based on their formal attributes, while experts focus on historic data related to the buildings to determine their value. According to Ledrut (1970), laypeople do not have precise knowledge of the historic data related to the main monuments of their city; they are important buildings simply because they are antique. Following the results of these studies, the levels of age of the built elements in the present research consider the details and ornament of the buildings, i.e., their “historic” appearance, and do not take into account the precise data on the construction year and the corresponding historical facts accompanying the buildings. Therefore, the dimension of age of the built elements was evaluated, considering the point of view of laypeople.

Regarding the biophilic level of the built elements, four specific dimensions are proposed. Two of them are related to the materials of the built elements in a place, while the other two are related to their shapes. The presence of greenery may be included as a dimension of biophilic design, as Purani and Kumar (2018) did. Nevertheless, to assess the effects that the built elements have on restoration, the proposed biophilic dimensions consider the built elements of the places exclusively.

#### Proportion of Built Elements with Biophilic Materials

This dimension (shortened in the following lines as biophilic materials) quantifies the built elements in a place possessing natural or natural-like materials, which present color variations, irregularities, and tactile textures. This dimension quantifies the built elements in a place possessing natural or natural-like materials, which present color variations, irregularities, and tactile textures. One of the most studied natural materials in interior spaces is wood, which reduces stress, tension, and fatigue, while increasing comfort and positive emotions (Burnard and Kutnar, 2015; Zhang et al., 2016, 2017). Kellert (2008, p. 7) emphasizes that people prefer authentic natural materials over artificial ones. Coeterier (2002, p. 115) indicates that while evaluating the beauty of a historical site, natural materials like brick and wood had a positive contribution: “They were experienced as living, warm, intimate, cosy and attractive; while modern materials like glass, steel and concrete were often called cold, dead, repellent or barren.” The high level of the biophilic materials dimension corresponds to places with many elements built with these types of materials. The intermediate level includes places in which some of their elements are made of biophilic materials. Environments in which there are no built elements presenting biophilic materials, or there is a small amount of them, pertain to the low level of this dimension.

#### Proportion of Built Elements with Non-biophilic Materials

The quantification of elements in a place with human-made materials lacking color variations and texture, such as the glass and steel mentioned above. Places with multiple built elements that do not present biophilic materials are included in the high level of this dimension. The intermediate level corresponds to the places with some non-biophilic built elements. The low level presents environments devoid of built elements with non-biophilic materials.

#### Proportion of Built Elements with Biophilic Shapes

This dimension (shortened as biophilic shapes) is the quantity of elements present in a place with curvilinear composite shapes with different scales of detail. The biophilic design element called “natural shapes and forms” by Kellert (2008) includes multiple qualities of the built elements that resemble the natural ones. Examples of those qualities are botanical and animal motifs, shapes lacking straight lines and right angles, shapes similar to shells, spirals, eggs, trees, etc. The latter attributes have been used to evaluate the biophilic qualities of built shapes in the study by Asim and Shree (2019). Evidence regarding human preference for curvilinear lines and shapes has been reviewed in Gómez-Puerto et al. (2016). Meanwhile, empirical research inquiring about beauty evaluations for curved and rectilinear interior spaces may be found in Vartanian et al. (2013, 2019). Besides curvilinearity, another characteristic that biophilic shapes may present is fractality: “ordered details arranged in a nested scaling hierarchy” (Salingaros and Masden, 2008). According to Taylor (2006), fractal shapes, whether those of natural elements, abstract paintings, or architecture, may reduce physiological stress. The three levels of the biophilic shapes in the present study relate to the varying degree of presence of the built elements with these specific characteristics.

#### Proportion of Built Elements with Non-biophilic Shapes

The quantity of elements in an environment that are simple cuboid volumes lacking details. Modern architecture buildings often possess non-biophilic shapes. In the words of Nia and Rahbarianyazd (2020, p. 66): “modern architecture is characterized by simple rectilinear shapes, plain undecorated walls, bald facades and, oftentimes, dull colors, using simple elementary shapes, lines, and forms as a basis for design.” Many existing modern buildings present those simple shapes. Nevertheless, modern buildings with curvilinear and more complex configurations also exist. The levels of the dimension of non-biophilic shapes correspond to the degree of presence of built elements lacking biophilic shapes in a place. As may be noticed, besides having one dimension for the biophilic level of the shapes of the built elements in the place and another for the materials, two separate dimensions for them are proposed. The latter allows a better evaluation of composite places such as those that present both elements with biophilic materials and elements with non-biophilic materials.

#### People Density

This dimension refers to the quantity of people present during the survey in relation to the extension of the place. The three levels correspond to crowded environments, places with a moderate amount of people, and places where no people or very few people were present. The photographs taken by the participants are the only evidence utilized for the evaluation of this dimension. High and low levels of people density in a place are found in Cozby (1973). Square meters per person and the number of people in the image are measured in Stamps (2015). Meanwhile, Hunter and Askarinejad (2015) do not measure people density but present four distinct categories related to people proximity in relation to the viewer (near, far, near and far, and no people present).

#### Street Visual Access

The degree to which the non-pedestrian roads visible in a place are close to the people who inhabit it. Environments in which visible streets are a few meters from the visited place correspond to the high level. This level of street visual access conveys the highest noticeability of parked and moving vehicles in the place. In the intermediate level, visible streets are around 50 m of distance or more. While the low level of this dimension is found in places where there are not streets close by and in places in which streets are not visible. It is a common practice in existing studies to compare natural scenes with “busy street” urban environments (Staats et al., 2016). There resides the importance of including environments with varying levels of street visual access in the present study.

Meanwhile, the environmental dimensions are specific and restrained evaluations of one attribute of a place (e.g., non-biophilic materials proportion), the psychological scales allow to obtain the impressions of the place as a whole (e.g., how preferred it is). It is hypothesized that if the subjects evaluated the places regarding the 12 dimensions, similar results to those of the experts could be obtained. The olfactory pleasantness and noise annoyance evaluated by the participants are also considered environmental qualities, as are the other 12 dimensions.

### Psychological Measures

Three outcome measures were considered in our study. These are the Mexican adaptation to the revised perceived restorativeness scale (RPRS) (Hartig et al., 1996; Martínez-Soto and Montero, 2008), the Spanish version of the profile of mood states (POMS) (Arce et al., 2000), and the stress and arousal adjectives checklist (SAACH) (King et al., 1983). We assessed restorative potential of the place, using RPRS. The scale components are derived from the theory of Kaplan of restorative environments (Kaplan and Kaplan, 1989). The RPRS is a 25-item scale that contains five subscales: being away (five items; e.g., “This place is a refuge from unwanted distractions”; α = 0.76), fascination (five items; e.g., “This place is fascinating”; α = 0.81), compatibility (five items; e.g., “It is easy to do what I want here”; α = 0.75), coherence (four items; e.g., “It is easy to see how things here are organized”; α = 0.75), and scope (four items; e.g., “I experience this place as very spacious”; α = 0.81) and two indicators of environmental preference (e.g., “I like this place”). The participants responded on an 11-point scale from 0 (*Nothing, does not apply to the experience described*) to 10 (*Completely, it does apply to the experience*). Ratings for 23 items (excluding environmental preference) were averaged to measure the perceived restorativeness of an individual (denominated as global restorative potential, GRP; Martínez-Soto et al., 2014a). Previous studies have shown that the RPRS is sensitive at the subscale level (Korpela and Hartig, 1996; Tenngart Ivarsson and Hagerhall, 2008). Similar to other studies, our research considers scalar scores of 0–4 (low), 5–7 (moderated), and 8–10 (high) to describe the restorative potential of the place (Berto, 2005; Martínez-Soto et al., 2014a). The mood states were measured, using the Spanish version of POMS (Arce et al., 2000), for which the participants rated 63 adjectives expressing mood states on a five-point Likert scale, ranging from 0 (*not at all*) to 4 (*very much*). The POMS consist of seven subscales, including vigor (eight items; α = 0.85), friendliness (seven items; α = 0.70), depression (14 items; α = 0.95), anger (12 items; α = 0.87), fatigue (seven items; α = 0.90), tension (eight items; α = 0.82), and confusion (seven items; α = 0.70). There are two options for assessing mood: the past week or now. In this study, the participants were asked to choose the right-now option. The SAACH instrument was used to measure situational stress. The Spanish version adapted by Ortega (2002) in Mexican population is an 18-item scale referring to the constructs of stress (seven items; α = 0.84), arousal (four items; α = 0.77), and exhaustion (four items; α = 0.72). The three subscales are rated, using a four-option response format: 1 (*yes definitely*) to 4 (*definitely not*). The odors of the city environment were measured through the sensory component of olfactory pleasantness and were rated using a single item “How pleasurable is the odor in this place?” 1 (*very unpleasant*) to 3 (*very pleasant*) (Baron and Kalsher, 1998; Hedblom et al., 2019). Finally, noise annoyance was evaluated through the item “How annoying is the noise in this place?” with the response options 1 (nothing) to 3 (very much). A description of the psychological measures used in our study and the corresponding rating scales are listed in Table 1.

**Table 1:** Psychological outcome and sensory dimensions.

**Variable**	**Type**	**Definition**	**Scale**
Being away	Restoration	Feeling of psychological or geographic detachment that promotes distraction and relaxation from directed attention.	0–10
Fascination	Restoration	A type of involuntary attention that allows directed attention to rest and recover.	0–10
Compatibility	Restoration	Adjustment between inclinations and personal purposes; as well as certain environmental limitations for the action of people.	0–10
Coherence	Restoration	It implies that the site has a structure and order and does not create confusion.	0–10
Scope	Restoration	Perception of extent that allows the exploration of the place.	0–10
Environmental preference	Restoration	Liking evaluation of a place.	0–10
Vigor	Mood	Alludes to a state of mind of euphoria and high energy.	0–4
Friendliness	Mood	It refers to a positive state of mind, of good disposition toward others.	0–4
Depression	Mood	It constitutes a depressive state, accompanied by a feeling of personal inadequacy.	0–4
Anger	Mood	Represents a feeling of anger and antipathy toward others.	0–4
Fatigue	Mood	Corresponds to a state of abatement (wear), inertia, and low level of energy.	0–4
Tension	Mood	It is defined by adjectives that reflect increases in tension skeletal muscle.	0–4
Confusion	Mood	It is characterized by disorientation and a multiplicity of thoughts.	0–4
Stress	Stress	Unpleasant psychological reactions reflected in restlessness, worry, tension, annoyance, and irritability.	1–4
Arousal	Stress	Psychological responses that people use to cope with stress, such as feeling active, vigorous, lively, and full of energy.	1–4
Exhaustion	Stress	Physiological and psychological reactions of people to stressful situations or to monotonous situations reflected in responses of fatigue and drowsiness.	1–4
Olfactory pleasantness	Sensory	Liking level of a place's smell.	1–3
Noise annoyance	Sensory	An unpleasant reaction to unwanted or strong sounds.	1–3

### Mobile Crowdsourcing Tools

**Figure 3:**
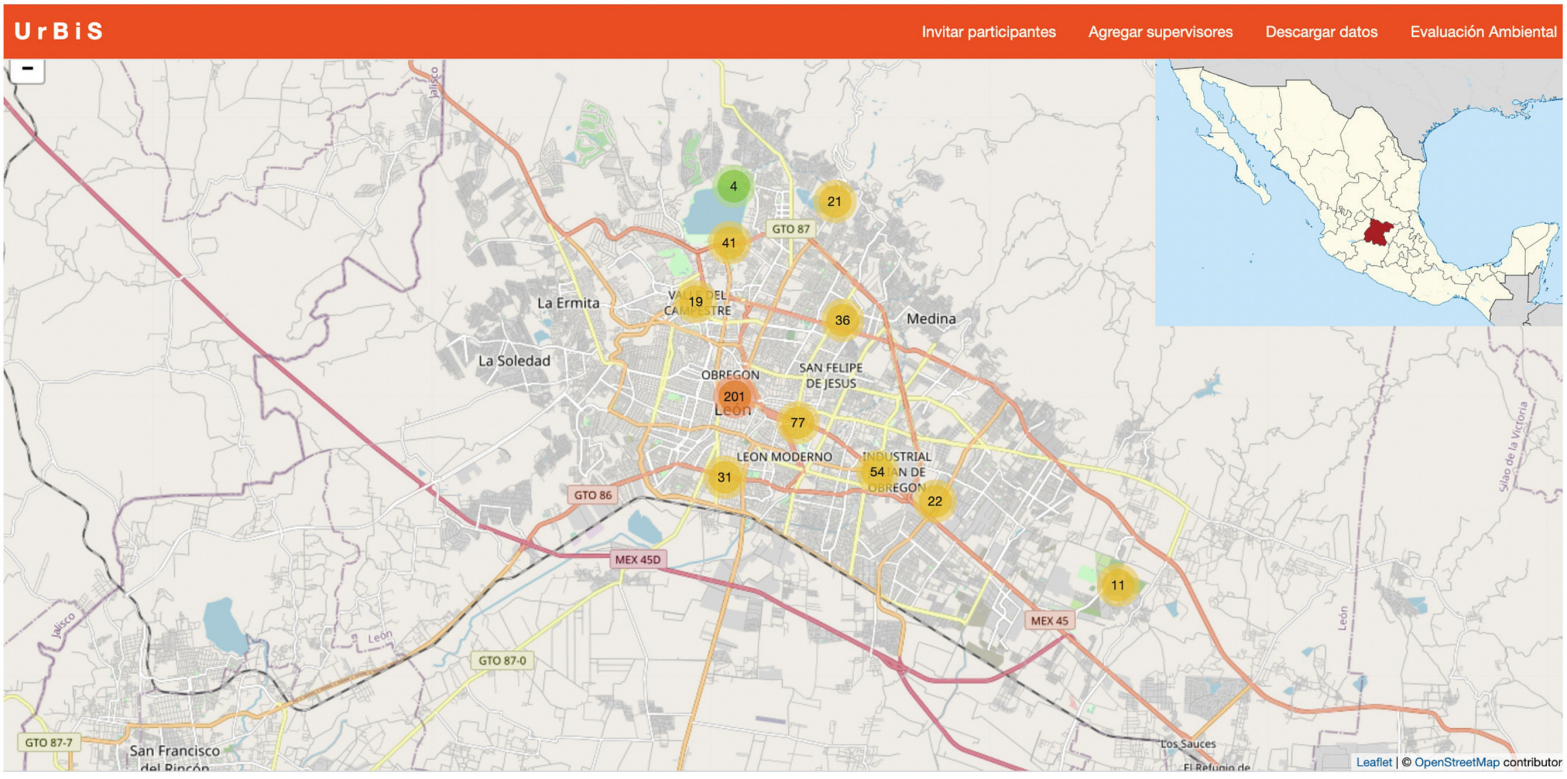
The UrBis platform map showing the points in which psychological and sensory data were collected in Leon, Mexico.

A mobile crowdsourcing platform (Ruiz-Correa et al., 2017), which includes a mobile application (called “UrBis”), enabled the administration of psychometric instruments in the field. UrBis allows app users to collect geolocalized multimedia data (photos, videos, audios, and text). UrBis users had to read and agree to the consent form to activate the app. The GPS location where each user completed the psychometric instruments was the only personal information collected. All mobile data collected remain in a Tier 3 data center at the National Supercomputer Center, sponsored by the Consejo Nacional de Ciencia y Tecnología (CONACYT). Data storing procedures followed a strict protocol that complies with the Mexican law concerning the security and privacy of contributions of users. Data minimization procedures enabled quantitative analysis requiring the safe use of GPS data. Minimization procedures include the use of GPS data with limited accuracy to prevent personal information disclosure. The UrBis app was developed for the Android platform (Figure 3). At the beginning of the survey, the participants were asked to complete a brief battery of demographic information (i.e., sex, age, the highest level of completed education, zip code for the location where they have lived for the longest, years of residence in the city, etc.). Correct completion of these data was required to proceed to the administration of the psychological scales.

### Data Collection Procedures

Two trained researchers visited the 65 spaces. Upon arriving at the places, they approached the site visitors, explained the study objectives, and invited them to participate. Two eligibility criteria were considered: (a) participants older than 16 years, and (b) city residents and visitants that are frequent users of the place (tourists were excluded from the study). All the participants first signed the informed consent and received instructions in collecting data, using UrBis mobile app. Then the investigator asked the participants to respond to a survey on sociodemographic information. Before answering the scales, the participants were asked to realize a 3-min free exploratory itinerary of the place to allow them to observe and appreciate the place without any constraint (de la Fuente Suárez, 2020, in press). During this experience, the participants were instructed to take photographs of the place. Then the participants were asked to answer the digitized versions of the RPRS, SAACH, and POMS to report the olfactory pleasantness of the place, noise annoyance, restorative potential, situational stress, and mood states they experienced when physically present in the explored setting (Jiang et al., 2019). The average response time was 20 min. Data were collected between January 2018 and July 2019, either during the week or the weekend and mainly on sunny days (*M* = 24.6°C; *SD* = 3.66). The surveys were conducted between 8–13 h and 18–20:30 h.

## Data Analysis

### Categorization of the Places

A multidimensional scaling (MDS) technique was used to categorize the 65 spaces based on 12 environmental qualities. The MDS is an exploratory data analysis that has been used in several studies relating environmental qualities and perceptions (Green, 1999; Coburn et al., 2019; de la Fuente Suárez, in press). In the present study, the MDS was realized in SPSS (PROXSCAL). The Euclidean distances between distinct places were used as dissimilarities.

### Survey Data Processing

Data from 470 collected surveys were included for analysis. Cronbach's alpha was computed for each scale and measure to verify their reliability. Statistical associations between the categories of the place and the psychological measures (restorative potential, moods, and stress) were conducted through non-parametric (Kruskal–Wallis ANOVA) analysis of variance due to deviations of sampled data from the Gaussian distribution. *Post-hoc* pairwise comparisons, using the Dunn–Bonferroni approach, were automatically conducted for any dependent variables for which the Kruskal–Wallis test was significant. The significance level was set to α = 0.05.

### Model Estimation Using Structural Equation Modeling

We conducted a confirmatory factor analysis and SEM (Rosseel, 2012; Li, 2016) to infer statistical associations among our measured environmental and psychological variables and five latent constructs: environmental qualities, restorative potential, positive and negative mood states, and situational stress (Figure 1). We used off-the-shelf *R* programming tools to implement SEM analyses (Rosseel, 2012). The following fit statistics were reported for the model estimation (Hoyle and Panter, 1995): the comparative fit index (CFI), the root mean square error of approximation (RMSEA), and the standardized root mean square residual (SRMR) (Hu and Bentler, 1999). Because chi-square is sensitive to sample size (Kline, 2011), with a high probability of being statistically significant with modest sample sizes (Iacobucci, 2010), we did not use it as a model fit measure. Instead, we followed the suggestions of Hu and Bentler (1999) and a set of cut-off criteria for a good fit (a CFI close to 0.95 or higher), RMSEA (close to 0.06 or lower), and SRMR (close to 0.08 or lower). Likewise, the percentages of variance explained (*R*^2^) in the outcomes latent variables were evaluated (Kline, 2011).

## Results

Reliabilities scales showed adequate to excellent (Kline, 2011) internal consistency indexes (Cronbach's α), ranging from 0.79 to 0.89 for RPRS dimensions, 0.73 to 0.83 for SAACH, and 0.67 to 0.90 to POMS subscales.

### Categorization of the Places According to Their Environmental Qualities

**Figure 4:**
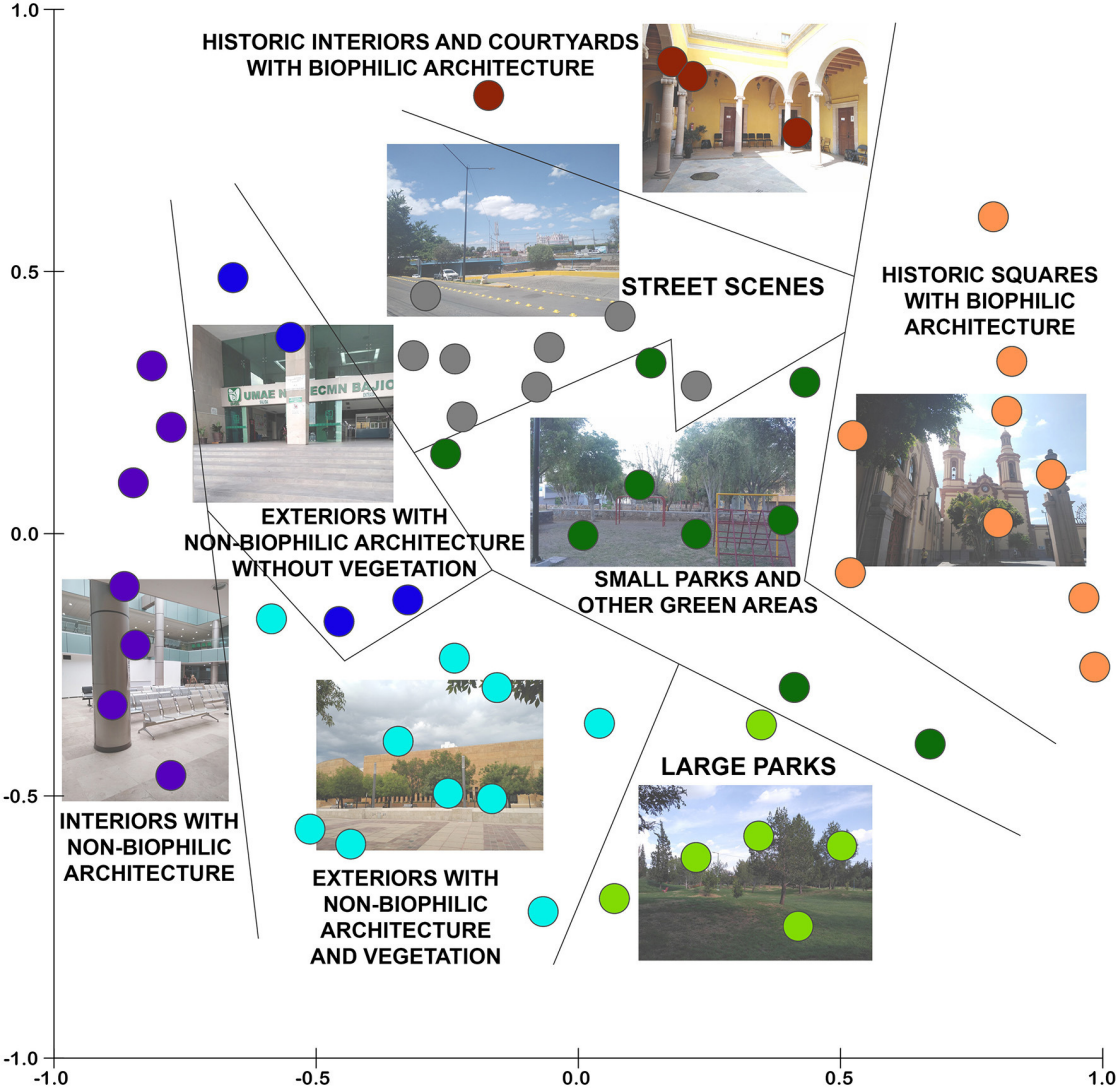
Multidimensional scaling (MDS) displaying the studied places in Leon, Mexico, grouped into eight categories (one photograph is presented for each category of place). The MDS is based on the similarities of the places regarding the 12 environmental dimensions.

By performing a MDS analysis (Figure 4), the 65 locations were grouped in eight categories of places, considering their similarity regarding the 12 environmental dimensions evaluated by the group of experts (Euclidean distances between data of the locations). The circles representing places in Figure 4 are closer to each other when they present more similarities regarding the 12 environmental dimensions. The resulting categories are (a) historic squares with biophilic architecture, (b) historic interiors and courtyards with biophilic architecture, (c) large parks, (d) small parks and other green areas, (e) street scenes, (f) exteriors with non-biophilic architecture and vegetation, (g) exteriors with non-biophilic architecture without vegetation, and (h) interiors with non-biophilic architecture. The utilization of MDS analysis allowed grouping the places in categories that emerged from the diagram on the basis of a set of environmental dimensions. The latter prevented that the categories of places were defined a priori and allowed for the discovery of categories that were more related to the particular nature of the studied places. In Table 2 may be noticed that six of the eight categories of places mainly correspond to exterior spaces (high level of exteriority). Large parks have the highest means of visibility and walkable surfaces of all types of places. This type of place also has the highest proportion of vegetation. The category of small parks and other green areas includes pocket parks, neighborhood parks, and median strips with vegetation. Compared with large parks, small parks are closer to streets. Regarding the street scenes, they are the places with the highest visual access to streets. Historic plazas with biophilic architecture possess the highest landmark salience, biophilic shapes, biophilic materials, and people density of all places. Meanwhile, the exteriors with non-biophilic architecture without vegetation present non-biophilic materials and non-biophilic shapes in their highest degree. Historic plazas and historic interiors are the types of places with the highest values of age of built elements. In opposition to the latter, the exteriors with non-biophilic architecture and vegetation, and the interiors with non-biophilic architecture are the environments with the newest built elements of all.

**Table 2:** Means and standard deviations per place category of the environmental dimensions evaluations given by the panel of experts.

	**Categories of place**							
	**Historic squares**	**Historic interiors and courtyards**	**Large parks**	**Small parks**	**Street scenes**	**Exteriors with non-bioph. arch. and vegetation**	**Exteriors with non-bioph. arch. without vegetation**	**Interiors with non-bioph. arch**.
**ENVIRONMENTAL QUALITIES** **Mean/STD**	**(11)**	**(4)**	**(7)**	**(9)**	**(11)**	**(10)**	**(4)**	**(9)**
Vegetation (0–2)	0.91/0.30	0.00/0.00	1.86/0.38	1.11/0.33	1.00/0.00	1.00/0.00	0.00/0.00	0.00/0.00
Exteriority (0–2)	2.00/0.00	0.25/0.50	2.00/0.00	2.00/0.00	2.00/0.00	1.40/0.52	1.75/0.50	0.00/0.00
Visibility (1–3)	2.18/0.40	1.00/0.00	2.57/0.53	2.11/0.33	2.36/0.50	2.10/0.74	2.25/0.96	1.33/0.71
Walkable surface (1–3)	2.36/0.67	1.25/0.50	2.86/0.38	2.11/0.60	1.00/0.00	1.80/0.63	1.75/0.96	1.33/0.50
Landmark salience (0–2)	1.91/0.30	0.75/0.96	0.43/0.53	0.33/0.71	0.45/0.69	1.20/0.79	0.75/0.96	0.00/0.00
Age of built elements (0–2)	2.00/0.00	2.00/0.00	0.71/0.49	1.11/0.33	1.00/0.00	0.20/0.42	0.50/0.58	0.44/0.53
Biophilic materials (0–2)	1.82/0.40	1.75/0.50	0.43/0.53	1.22/0.67	0.09/0.30	0.40/0.52	0.00/0.00	0.11/0.33
Non-biophilic materials (0–2)	0.18/0.40	0.25/0.50	0.43/0.53	0.56/0.73	1.73/0.47	1.60/0.52	2.00/0.00	1.89/0.33
Biophilic shapes (0–2)	1.82/0.40	1.00/0.00	0.43/0.53	0.33/0.50	0.18/0.40	0.40/0.52	0.00/0.00	0.00/0.00
Non-biophilic shapes (0–2)	0.18/0.40	1.00/0.00	0.43/0.53	1.11/0.78	1.64/0.50	1.60/0.52	2.00/0.00	2.00/0.00
People density (0–2)	1.00/0.89	0.00/0.00	0.43/0.79	0.11/0.33	0.18/0.40	1.20/0.79	0.00/0.00	1.22/0.67
Street visual access (0–2)	1.18/0.60	0.00/0.00	0.29/0.49	1.56/0.53	2.00/0.00	0.30/0.48	1.00/0.82	0.22/0.44

### Restorative Potential (GRP) Across the Categorized Settings

A Kruskal–Wallis test showed that the category of place significantly affects the GRP [(H7) = 122.86, *p* < 0.00; η^2^ = 0.25]. The Bonferroni *post-hoc* method, following the Kruskal–Wallis test, found that the eight categories of places differed in their GRP. Historic squares (mean rank = 219.74) had a higher restorative potential than the small parks (mean rank = 215.76; *p* < 0.05) and the interiors with non-biophilic architecture (mean rank = 115.82; *p* < 0.00), which, in turn, differ significantly from each other (*p* < 0.05). In overall, large parks (mean rank = 340.93) had a higher GRP than exteriors with non-biophilic architecture and vegetation (mean rank = 268.90*; p* < 0.00), small parks (mean rank = 215.76*; p* < 0.00), street scenes (mean rank = 180.43*; p* < 0.00), interiors with non-biophilic architecture (mean rank = 115.82*; p* < 0.05), and exteriors with non-biophilic architecture without vegetation (mean rank = 136.35*; p* < 0.05). Both non-biophilic interiors and exteriors with non-biophilic architecture without vegetation differ, respectively, with exteriors with non-biophilic architecture and vegetation (mean rank = 268.90; *p* < 0.05). Finally, historic interiors (mean rank = 285.47) had a higher GRP than interiors with non-biophilic architecture (mean rank = 115.82; *p* < 0.05) (Tables 3, 4).

**Table 3:** Mean rank scores of the GRP, mood states, situational stress, and the sensory dimensions of the categories of place as assessed by the participants of the study.

	**Categories of place**							
**Psychological** ** and sensory** ** dimensions**	**Historic squares**	**Historic interiors and courtyards**	**Large parks**	**Small parks**	**Street scenes**	**Exteriors with non-bioph. arch. and vegetation**	**Exteriors with non-bioph. arch. without vegetation**	**Interiors with non-bioph. arch**.
**Mean/STD ** ** Mean rank**	**(*n* = 110)**	**(*n* = 18)**	**(*n* = 89)**	**(*n* = 45)**	**(*n* = 47)**	**(*n* = 91)**	**(*n* = 13)**	**(*n* = 57)**
GRP (0–10)	7.39/1.73	8.36/0.87	8.77/1.13	7.31/1.93	7.08/1.48	8.02/1.44	5.97/2.23	5.59/2.29
	219.74	285.47	340.93	215.76	180.43	268.90	136.35	115.82
Vigor (0–4)	3.38/0.71	3.39/0.85	3.30/0.68	3.53/0.86	3.47/0.65	3.27/0.70	2.92/0.76	3.21/0.77
	242.90	247.86	228.96	275.1	261.19	222.03	168.81	211.79
Friendliness (0–4)	3.62/0.67	3.61/0.69	3.54/0.67	3.64/0.80	3.64/0.79	3.51/0.76	3.08/0.64	3.58/0.75
	244.65	246.33	230.74	255.83	243.33	226.41	151.69	232.8
Depression (0–4)	1.25/0.62	1.11/0.32	1.10/0.33	1.16/0.42	1.15/0.41	1.20/0.47	1.38/0.50	1.14/0.35
	242.46	226.44	221.97	232.29	230.96	239.87	289.08	233.14
Anger (0–4)	1.28/0.60	1.11/0.32	1.15/0.38	1.16/0.36	1.09/0.28	1.27/0.55	1.23/0.43	1.30/0.59
	243.28	218.28	224.14	228.39	212.36	245.28	245.50	250.46
Fatigue (0–4)	1.45/0.74	1.56/0.70	1.20/0.48	1.24/0.43	1.21/0.54	1.32/0.55	1.38/0.50	1.53/0.65
	247.86	275.28	209.07	223.77	206.65	233.30	254.88	272.48
Tension (0–4)	1.55/0.72	1.56/0.61	1.36/0.50	1.38/0.61	1.40/0.49	1.57/0.63	2.00/0.70	1.67/0.69
	237.81	226.94	209.11	206.62	220.23	248.26	324.00	263.46
Confusion (0–4)	1.61/0.70	1.56/0.51	1.43/0.56	1.36/0.52	1.34/0.56	1.60/0.59	1.77/0.72	1.68/0.57
	245.28	247.33	215.03	200.46	194.83	252.05	277.62	270.03
Stress (1–4)	1.31/0.55	1.28/0.46	1.13/0.37	1.22/0.42	1.40/0.61	1.36/0.56	1.85/0.68	2.08/0.76
	229.45	229.58	195.48	217.17	247.83	242.77	332.46	281.52
Arousal (1–4)	3.41/0.72	3.56/0.61	3.55/0.60	3.53/0.69	3.57/0.58	3.44/0.67	3.23/0.72	3.26/0.83
	228.48	251.31	249.96	252.07	253.89	231.37	192.92	209.53
Exhaustion (1–4)	1.65/0.82	1.89/0.75	1.00/0.73	1.69/0.84	1.68/0.83	1.71/0.86	2.08/0.64	2.09/0.76
	221.03	265.44	215.66	226.28	225.5	229.69	300.19	294.99
Olfactory	2.10/0.66	2.39/0.50	2.76/0.45	2.49/0.50	2.09/0.50	2.60/0.59	2.15/0.37	2.12/0.60
pleasantness (1–3)	184.4	231.89	314.29	253.49	173.46	283.49	180.88	184.96
Noise annoyance (1–3)	1.71/0.61	2.06/0.53	1.21/0.41	1.40/0.53	1.72/0.61	1.49/0.56	2.15/0.80	1.91/0.62
	259.31	326.78	159.04	197.59	262.12	217.17	327.5	296.38

**Table 4:** Comparisons of the GRP scores across the categories of place (*N* = 470, pairwise comparison *p*-values).

**Categories of place**	**Historic squares**	**Historic interiors and courtyards**	**Large parks**	**Small parks**	**Street scenes**	**Exteriors with non-bioph. arch. and vegetation**	**Exteriors with non-bioph. arch. without vegetation**	**Interiors with non-bioph. arch**.
Historic squares	–							
Historic interiors and courtyards	ns	–						
Large parks	0.00	ns	–					
Small parks	0.05	ns	ns	–				
Street scenes	ns	ns	0.05	0.05	–			
Exteriors with non-bioph. arch. and vegetation	0.00	ns	ns	ns	0.05	–		
Exteriors with non-bioph. arch. without vegetation	ns	ns	0.05	ns	ns	ns	–	
Interiors with non-bioph. arch	ns	ns	0.00	ns	0.05	0.00	ns	–

### Mood States Across the Categorized Settings

The Kruskal–Wallis *H*-test revealed that there were statistically significant differences between the mood state of fatigue and the type of environment [(H7) = 20.75, *p* < 0.00; η^2^ = 0.03]. Furthermore, the *post-hoc* analysis shows higher scores of fatigue in interiors with non-biophilic architecture (mean rank = 272.48) compared with large parks (mean rank = 209.07) and street scenes (mean rank = 206.65) (adjusted *p* < 0.05, respectively). For tension, significant differences emerged with respect to the type of environment [(H7) = 19.25, *p* < 0.00; η^2^ = 0.02]. *Post-hoc* analysis revealed higher scores of tension for exteriors with non-biophilic architecture without vegetation (mean rank = 324) vs. large parks (mean rank = 209.11) (adjusted *p* < 0.05, respectively). In confusion, the mean scores were found to have statistically significant differences between the environments according to the Kruskal–Wallis *H*-test [(H7) = 20.77, *p* < 0.00; η^2^ = 0.03]. *Post-hoc* pairwise comparisons revealed statistically differences between interiors with non-biophilic architecture (mean rank = 270.03) and street scenes (mean rank = 194.83) (adjusted *p* < 0.05). Finally, no statistical differences were observed across the eight categories of settings with respect to the mood states of vigor [(H7) = 14.43, *p* = 0.04; η^2^ = 0.01], friendliness [(H7) = 8.68, *p* = 0.27; η^2^ = 0.00], depression [(H7) = 9.18, *p* = 0.24; η^2^ = 0.00], and anger [(H7) = 8.93, *p* = 0.25; η^2^ = 0.00].

### Situational Stress Across the Categorized Settings

The Kruskal–Wallis ANOVA-test revealed that the type of environment significantly affects the stress measures [(H7) = 35.82, *p* < 0.00; η^2^ = 0.06]. Furthermore, the *post-hoc* analysis shows that large parks (mean rank = 195.48) were experienced with lower levels of stress compared with interiors with non-biophilic architecture (mean rank = 281.52; adjusted *p* < 0.00) and exteriors with non-biophilic architecture without vegetation (mean rank = 332.46; adjusted *p* < 0.05). Likewise, small parks (mean rank = 217.17) and interiors with non-biophilic architecture (mean rank = 281.52) were perceived with lower scores of stress than exteriors with non-biophilic architecture without vegetation (mean rank = 332.46) (adjusted *p* < 0.05). Concerning exhaustion, the non-parametric test shows statistically significant differences between the type of environment [(H7) = 21.82, *p* < 0.00; η^2^ = 0.03]. The *post-hoc* analysis revealed that compared with large parks (mean rank = 215.66) and historic squares (mean rank = 221.03), interiors with non-biophilic architecture were perceived with higher scores of exhaustion (mean rank = 294.99) (adjusted *p* < 0.05, respectively). No statistical differences were observed across the eight categories of settings with respect to the arousal dimension [(H7) = 8.31, *p* = 0.30; η^2^ = 0.03].

### Olfactory Pleasantness and Noise

The mean score of the perceived environmental quality of olfactory pleasantness measured by the item “How pleasurable is the odor in this place?” was found to have statistical significant differences between the groups of environments [(H7) = 95.57, *p* < 0.00; η^2^ = 0.19]. Subsequent *post-hoc* pairwise comparisons (Table 5) revealed that, overall, large parks were perceived with higher levels of olfactory pleasantness (mean rank = 312.29) than street scenes (mean rank = 173.46; adjusted *p* < 0.05), exteriors with non-biophilic architecture without vegetation (mean rank = 180.88; adjusted *p* < 0.05), interiors with non-biophilic architecture (mean rank = 184.96; adjusted *p* < 0.00) and historic squares (mean rank = 184.4; adjusted *p* < 0.00). Likewise, exteriors with non-biophilic architecture and vegetation (mean rank = 283. 49) were perceived with higher scores of olfactory pleasantness compared with interiors with non-biophilic architecture (mean rank = 184.96; adjusted *p* < 0.00), historic squares (mean rank = 184.4; adjusted *p* < 0.00), and street scenes (mean rank = 173.46; adjusted *p* < 0.00). In the same way, street scenes were perceived with lower scores of olfactory pleasantness compared with small parks (mean rank = 253.49; adjusted *p* < 0.00). Finally, small parks were perceived with higher values of olfactory pleasantness than historic squares (mean rank = 184.4; adjusted *p* < 0.05).

**Table 5:** Comparisons of olfactory pleasantness scores across the settings (*N* = 470, pairwise comparison *p*-values).

**Categories of place**	**Historic squares**	**Historic interiors and courtyards**	**Large parks**	**Small parks**	**Street scenes**	**Exteriors with non-bioph. arch. and vegetation**	**Exteriors with non-bioph. arch. without vegetation**	**Interiors with non-bioph. arch**.
Historic squares	–							
Historic interiors and courtyards	ns	–						
Large parks	0.00	ns	–					
Small parks	0.05	ns	ns	–				
Street scenes	ns	ns	0.05	0.05	–			
Exteriors with non-bioph. arch. and vegetation	0.00	ns	ns	ns	0.05	–		
Exteriors with non-bioph. arch. without vegetation	ns	ns	0.05	ns	ns	ns	–	
Interiors with non-bioph. arch	ns	ns	0.00	ns	0.05	0.00	ns	–

Mean scores related to noise annoyance (“How annoying is the noise in this place?”), with the response options 1 (*nothing*) to 3 (*very much*), were statistically different across the environmental categories according to the non-parametric Kruskall–Wallis *H*-test results [(H7) = 80.17, *p* < 0.00; η^2^ = 0.15]. The *post-hoc* analysis revealed (Table 6) that large parks (mean rank = 159) were self-evaluated with lower noise than exteriors with non-biophilic architecture (with and without vegetation) (mean rank = 217.17 and 327.5; adjusted *p* < 0.05, respectively). In the same manner, large parks were perceived as less noisy than the historic squares (mean rank = 259.31; adjusted *p* < 0.00) and interiors with non-biophilic architecture (mean rank = 296.38; adjusted *p* < 0.00). After large parks, small parks were perceived with lower levels of noise annoyance compared to exteriors with non-biophilic architecture without vegetation (mean rank = 327.35; adjusted *p* < 0.05), historic interiors and courtyards (mean rank = 326.78; adjusted *p* < 0.05), and interiors with non-biophilic architecture (mean rank = 296.38; adjusted *p* < 0.05). Finally, both historic interiors and interiors with non-biophilic architecture were perceived with higher scores of noise annoyance compared to exteriors with non-biophilic architecture and vegetation (adjusted *p* < 0.05, respectively) (Table 6).

**Table 6:** Comparisons of noise scores across the places (*N* = 470, pairwise comparison *p*-values).

**Categories of place**	**Historic squares**	**Historic interiors and courtyards**	**Large parks**	**Small parks**	**Street scenes**	**Exteriors with non-bioph. arch. and vegetation**	**Exteriors with non-bioph. arch. without vegetation**	**Interiors with non-bioph. arch**.
Historic squares	–							
Historic interiors and courtyards	ns	–						
Large parks	0.00	ns	–					
Small parks	ns	0.05	ns	–				
Street scenes	ns	ns	ns	ns	–			
Exteriors with non-bioph. arch. and vegetation	ns	0.05	0.05	ns	ns	–		
Exteriors with non-bioph. arch. without vegetation	ns	ns	0.00	0.05	ns	ns	–	
Interiors with non-bioph. arch	ns	ns	0.00	0.05	0.05	0.05	ns	–

### Structural Equation Modeling

**Figure 5:**
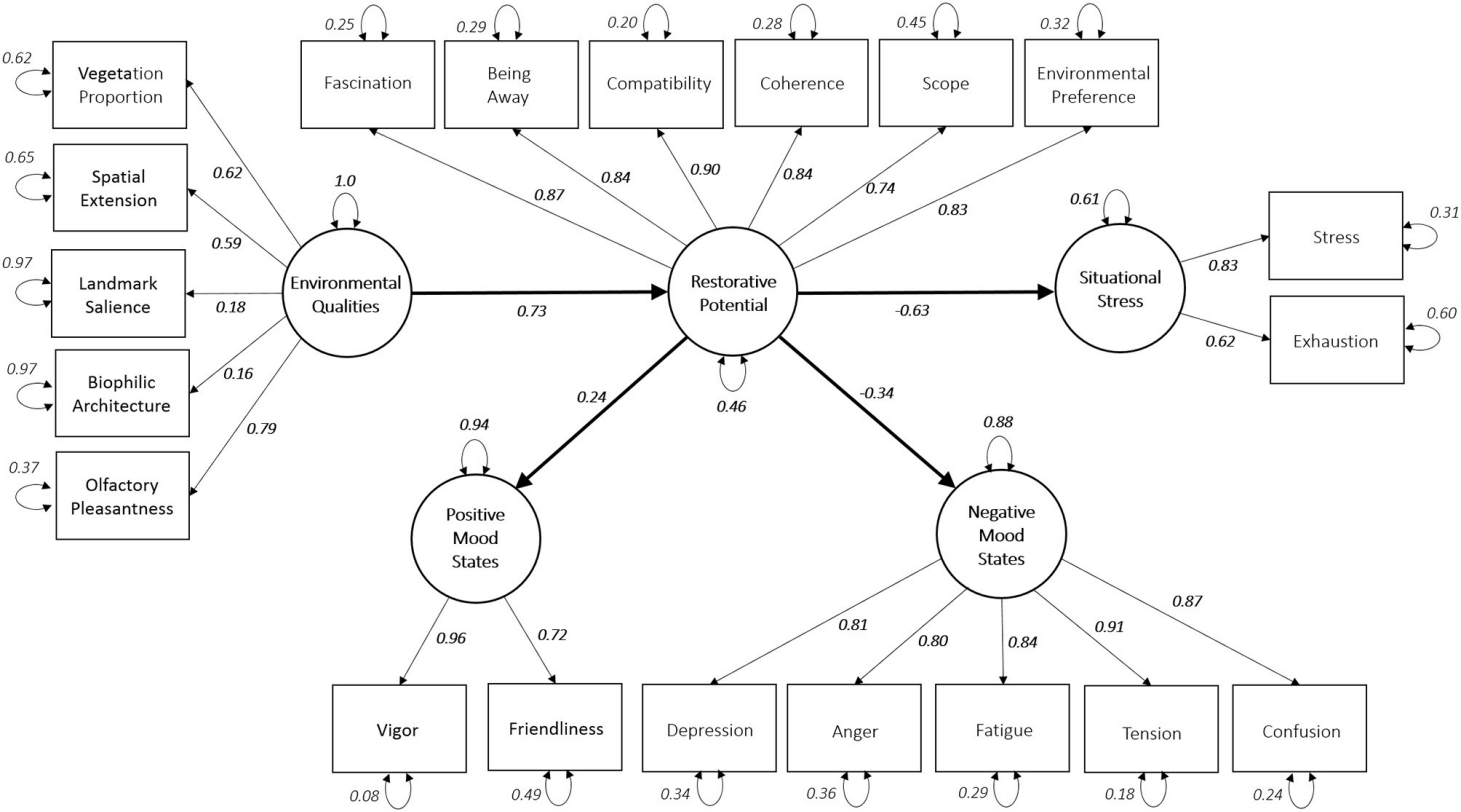
Structural equation modeling showing the relations between the measured variables and the five latent variables: environmental qualities, restorative potential (including all the EPRA-R dimensions), positive mood states, negative mood states, and situational stress. Model fit indexes, CFI = 0.96, RMSEA = 0.05, SRMR = 0.07.

Lavaan software package (Rosseel, 2012) enabled statistical inference of our structural equation model (Figure 5). The package reports a CFI of 0.96, an SRMR value of 0.07, and an RMSEA equal to 0.05 (with *p* < 0.001) with a confidence interval of (0.42, 0.53), suggesting a moderately good model fit. For our data set, the package uses the maximum likelihood estimation with robust standard errors and a mean and variance adjusted test statistics. Table 7 summarizes the descriptive statistics of the model, while the covariance matrix of the latent variables is shown in Table 8. The structural equation allowed us to characterize five latent variables (environmental qualities, restorative potential, situational stress, positive mood states, and the negative mood states) that link the psychological and environmental variables considered in the study. The environmental qualities variable considers all the physical, architectural, and sensory aspects that result in restoration. Such a variable is linked to the following five dimensions: vegetation, spatial extension, landmark salience, biophilic architecture, and olfactory pleasantness. Spatial extension groups the exteriority, visibility, and walkable surface variables. These three variables are significantly correlated. The spatial extension variable is related to the depth dimension proposed by Ulrich (1977, p. 5), which is motivated by the observation that “[.] if a man has a predilection for exploring and actively seeking information, then spaciousness or depth should be favored. The presence of space signals opportunities for entering a scene and garnering new information…”

**Table 7:** Descriptive statistics of the factors included in the model.

**Factor**	**Measures and value ranges**	***Mean***	***SD***
Environmental qualities	Vegetation proportion (0–2)	1.02	0.63
	Spatial extension (2–8)	6.06	1.88
	Landmark salience (0–2)	0.94	0.87
	Biophilic architecture (0–10)	4.65	3.44
	Olfactory pleasentness (1–3)	2.37	0.62
Restorative potential	Fascination (0–10)	7.42	2.30
	Being Away (0–10)	7.31	2.45
	Compatibility (0–10)	7.49	2.12
	Coherence (0–10)	8.21	1.80
	Scope (0–10)	7.53	2.07
	Environmental preference (0–10)	7.44	2.60
Situational stress	Stress (1–4)	1.34	0.59
	Exhaustion (1–4)	1.73	0.81
Negative mood states	Depression (0–4)	1.18	0.46
	Anger (0–4)	1.22	0.50
	Fatigue (0–4)	1.34	0.60
	Tension (0–4)	1.51	0.63
	Confusion (0–4)	1.53	0.61
Positive mood states	Vigor (0–4)	3.34	0.73
	Friendliness (0–4)	3.57	0.72

**Table 8:** Latent variables correlation matrix.

	**Environmental qualities**	**Restorative potential**	**Situational stress**	**Negative mood states**	**Positive mood states**
Environmental qualities	1.00				
Restorative potential	0.73	1.00			
Situational stress	−0.46	−0.63	1.00		
Negative mood states	−0.25	−0.34	0.72	1.00	
Positive mood states	0.18	0.24	−0.50	−0.35	1.00

The biophilic architecture variable is a general score of a place that reaches its maximum value when the age of the built elements and the biophilic materials and shapes are in the high levels, while the non-biophilic materials and shapes are in the low levels. Therefore, biophilic architecture corresponds to the sum of the values of age of the built elements, biophilic materials, biophilic shapes, non-biophilic materials (inverted), and non-biophilic shapes (inverted). All dimensions included in this composite variable are statistically correlated. The inclusion of people density, street visual access, noise annoyance, and arousal variables hindered the model fit results. The final model did not consider these variables (Hatcher, 1994).

Our structural model shows that both situational stress and the positive and negative mood states indirectly depend on the latent environmental quality variable through the restorative potential variable. The directional associations are illustrated through single-headed straight arrows with standardized and significant path coefficients, indicating the strength of associations. Size effects are described according to an absolute standardized direct result where a value smaller than 0.1 is considered as an indication of a small effect, a value around 0.30 is regarded as an indicator of a typical or medium impact, and a value greater than 0.5 is considered as an indicator of a more significant effect (Bollen, 1989). Referred *R* squares (*R*^2^) of the endogenous variables represent the percentage of the variance of each endogenous variable (Kline, 2011).

The stress variable has the most significant negative and direct path coefficient with the restorative potential variable, with a large-sized effect (−0.63) and 39% of explained variance. The negative mood states variable has a moderate impact (−0.34), with 12% explained variance. The positive mood states variable has an average effect (0.24) and 6% of the explained variance with a moderate effect size. The environmental qualities latent variable directly enhances (with a path coefficient of 0.73) the restorative potential (Figure 5), which, in turn, has negative path coefficients with stress (−0.63), negative mood states (−0.34), and a positive path coefficient with positive mood states (0.24). Figure 5 shows an indirect negative association between the environmental quality variable, the situational stress (−0.46), and negative mood states (−0.25) through restorative potential. Likewise, the SEM also shows that the positive mood states involve the indirect linear effects of environmental qualities through restorative potential (0.18) (see Table 8). In summary, an increase in the environmental qualities values increases the restorative potential and the positive mood states (with a combined path coefficient of 0.17). It decreases the situational stress and negative mood states.

## Discussion

This study investigated the restorative impact of biophilic design-based features and other environmental and sensory qualities of exterior and interior spaces on mood states and psychological stress in Leon, Mexico. This section discusses the findings of our study and future directions in the context of our research questions.

### Q1. How Can Interior and Exterior Spaces Be Categorized According to Their Environmental Qualities?

A MDS technique was used to categorize the 65 spaces based on 12 environmental qualities rated by a panel of experts. The use of MDS analysis based on such evaluations allowed for generating specific categories of real places found in a Mexican city. The present study focused on the significant differences regarding the restorative qualities of the categories of place having different magnitudes of the environmental dimensions as they naturally occur. The holistic selection of the categories of place played a role in the significant differences between the categories regarding their restorative potential. Other environmental dimensions, e.g., related to the use or function of the spaces, should be inquired in future studies.

### Q2. Do the Categories of Place Make a Difference in the Restorative Potential, Stress, and Mood States as Perceived by People?

#### Restorative Potential and Environment

Comparative analysis shows that the type of categorized places affects the perceptions of people of restorative (global) potential, negative mood states (fatigue, tension, and confusion), and situational stress (stress and exhaustion). The category of large parks, which has the highest amount of vegetation of all categories, is also the one with the highest scores of restorative potential, being consistent with previous studies (Giuseppe et al., 2013; Lorenzo et al., 2016; Pasanen et al., 2018a; Cheon et al., 2019; Kang and Kim, 2019). Meanwhile, small parks present moderate levels of restorative potential, suggesting that the spatial extension and the higher vegetation proportion of large parks play a vital role in promoting a higher restoration. In addition, our data confirm that street scenes with moderate vegetation levels are related to restorative experiences (Masoudinejad and Hartig, 2020). According to the results, environments with lower scores of restorative potential (e.g., interiors with non-biophilic architecture) do not present the positive attributes labeled here as environmental dimensions. The latter situation limits the opportunities to evoque restorative experiences (Evans and McCoy, 1998; Lindal and Hartig, 2013; Masoudinejad and Hartig, 2020). Historic squares and small parks are categories of places with moderate restorative potentials. In the historic squares, the physical design attributes, such as landmark salience and biophilic architecture, could be working together with the cultural aspects related to the place (i.e., its historical value) to contribute to their restorative potential. In this study, historic squares are touristic places where social and leisure activities are present, adding a situational and social value that positively impacts their restorative potential (Scopelliti and Giuliani, 2004; Nordh et al., 2011). The findings agree with the notion that both the biophilic qualities of the buildings and the leisure and culture possibilities offered by the spaces contribute to the restorative value of a place (Herzog and Shier, 2000; Herzog et al., 2010; Weber and Trojan, 2018; Coburn et al., 2019). The typical dichotomy between the commonly low restorative built environments and the high restorative natural environments (Verlade et al., 2007) needs a further re-examination when considering the biophilic, cultural, and social aspects of places that also promote restorative outcomes. It is remarkable that historic interiors and courtyards with biophilic architecture are the second category of settings with the highest restorative potential. The cultural and biophilic character of the latter spaces makes a difference in the restorative potential compared to those interior scenarios lacking these qualities. Our findings agree with other studies in which the restorative potential of cultural environments is similar to that of natural environments (Xu et al., 2018). In the future studies, the categories of place should be analyzed, considering the distinct dimensions that compose the restorative potential, i.e., being away, extent, fascination, and compatibility.

#### Mood States, Situational Stress, and Environment

Assuming that the restorativeness scores may explain the mood changes in the studied environments (Van den Berg et al., 2003; Stigsdotter et al., 2017), the outcomes showed that exposure to high and moderate restorative environments is associated with mood influences related to lower levels of fatigue, tension, and confusion (e.g., Lee et al., 2017). The scores reveal that exposure to large parks and street scenes reduced some negative moods (with lower magnitude effects) but had little impact on positive feelings. These smaller effects could be related to negative attitudes toward the city (Félonneau, 2004; Galindo and Hidalgo, 2005; Gidlow et al., 2016). Regarding stress, large and small parks are less stressful than interiors and exterior spaces with non-biophilic architecture without vegetation. Concerning exhaustion, large parks and historic squares were less exhausting than the interiors with non-biophilic architecture. There were no influences of the environmental categories on psychological arousal states. Our results support the empirical findings on affective restoration outcomes related to vegetal cover (Huang et al., 2020) in large recreational parks (Kajosaari and Pasanen, 2021) and in studies without experimental interference (Hazer et al., 2018) related to lower perceived stress on such spaces (Hunter et al., 2019). Based on the preceding observations, the following question arises: Are there other architectural or environmental qualities or other place categories that present stronger relations with particular mood states and situational stress? All the places studied here correspond to environments inhabited or visited by people on a quotidian basis, whether for leisure or other purposes. Those spaces possess a moderate to low-noise level and people density and are considered safe spaces. Nevertheless, several types of environments present in contemporary cities were not included in the study, such as the spaces around and inside metro stations, bus stops, or multistory car parks. In the future studies, including the latter spaces may increase the chances that the categories of places result in more significant differences in mood states and situational stress.

#### Sensory Qualities and Environment

The results suggest that the spaces with nature: large parks, exteriors with non-biophilic architecture and vegetation, and small parks were perceived with higher levels of olfactory pleasantness compared with the rest of the categories. Our data indicate that the olfactory experiences with green spaces are as meaningful as the visual contact with nature to promote restorative experiences. Further field studies need to be developed to clarify these results (e.g., Henshaw, 2014). The results show that green spaces present lower scores on noise annoyance within the set of environmental categories. Our data indicate that lower noise annoyance scores in green areas (e.g., large parks) are related to higher scores of perceived restorativeness, which is in line with other studies (e.g., Shaw et al., 2015). A limitation of our study that should be inquired further is that it only considered the negative facet of environmental sound that presumably constrains the restorative experiences, leaving aside the role of nature sounds that enhance the restorativeness of the place and its psychological effects. Meanwhile, the participants were fully immersed in the places while answering the scales; the panel of experts depended on the photographs of the places to evaluate the environmental dimensions. The latter is a limitation of the present study to overcome in the future studies.

### Q3. How Can Statistical Dependencies Between the Environmental Qualities, the Restorative Potential, Stress, and Mood States Be Quantified Through a Structural Equations Model?

As expected, the environmental qualities had an indirect path through the restorative potential. According to the model in Figure 5, there was a direct and positive effect between environmental qualities and restorative potential. As observed, the construct environmental qualities conformed by the dimensions of (a) vegetation, (b) spatial extension, (c) landmark salience, (d) biophilic architecture, and (e) olfactory pleasantness share a significant percentage of explained variance with the restorative potential variable, suggesting a relative capability to predict the restorative potential of the place. Our results confirm that the experience of spending time in environments with restorative qualities reduces the perception of psychological stress and negative moods and improves positive mood states, with moderate to high size effects. The relation between affective and stress states reported are congruent with both field and experimental studies on built restorative environments (Johansson et al., 2011; Lindal and Hartig, 2013; Stigsdotter et al., 2017; Hazer et al., 2018; Pasanen et al., 2018b; Hedblom et al., 2019; Hunter et al., 2019; Truong et al., 2019; Kajosaari and Pasanen, 2021; Subiza-Pérez et al., 2021) and related studies on restorative influences of indoor and outdoor biophilic environments (Hoyle et al., 2017; Yin et al., 2018, 2020; Ortegón-Cortázar and Royo-Vela, 2019; Roskams and Haynes, 2020). Further studies of replication with direct and indirect measures of the studied environmental qualities are needed to confirm its restorative influences, considering more robust measures of restorative outcomes (e.g., cognitive and physiological restoration indexes).

### Q4. What Design Guidelines May Be Proposed on the Basis of the Findings of This Study?

Planning a space by paying attention to the spatial extension, biophilic materials and shapes, vegetation, and pleasant smells guarantees the restoration. Following results of this study, it may seem that architects and urban planners should confront the unsolvable problem of designing built environments for people that inherently prefer the natural and despise the non-biophilic built environments. Not all environments are in contact with nature or permit the maintenance of vegetation. There resides the importance of the inclusion of architectural elements being designed with biophilic principles since they solve the aversion to the non-biophilic built elements and allow environmental restoration. It is important to remark that, in this study, the places with higher proportions of biophilic materials and shapes are those with old-style architecture (higher age of the built elements). Other studies could consider the inclusion of historic places without biophilic architecture (e.g., simple antique buildings) and contemporary places with biophilic architecture (e.g., parametric architecture works). In that manner, it would be clearer if what makes a place restorative is its historic value, independently of its biophilic qualities, or if the biophilic materials and shapes are what make a place restorative, independently of its historic value. Four dimensions related to biophilic shapes and materials were presented. More research is needed in which even more specific dimensions are inquired. The dimension of biophilic shapes could be divided into naturalness, curvilinearity, and complexity. Meanwhile, the dimension of biophilic materials could be subdivided into the naturalness of the material and the distinct tactile qualities of the texture.

## Contributions

This study makes important contributions to both the theory and practice of restorative and biophilic design. This research extends the empirical, methodological, and practical knowledge about the issues related to the psychological benefits of the built restorative spaces. Most of the research on restorative built environments has been focused on outdoor spaces, while research on biophilic spaces focuses on indoor spaces. The importance of the present study resides in examining a diversity of places with different environmental characteristics (including those with biophilic design) and restorative outcomes, expanding thus the knowledge of the psychological benefits of built restorative spaces. Likewise, this investigation incorporates both visual and non-visual sensory dimensions on restorative experiences, which resulted significantly relevant. To our knowledge, this is the first study where the place perceived restorativeness is linked with its olfactory pleasantness. Furthermore, an ecological criterion of validity was guaranteed by the method of place categorization developed in our study, which emphasizes the singular and natural characteristics of the places. Lastly, our study added practical recommendations since the psychological and environmental data that consider the ecological, spatial, architectural, cultural, and social traits immersed in real places will be of utility in designing and reconfiguring healthy environments.

## Data Availability Statement

The raw data supporting the conclusions of this article will be made available by the authors, without undue reservation.

## Ethics Statement

This study involving human participants was reviewed and received ethical approval by the Comité Institucional de Bioética en la Investigación de la Universidad de Guanajuato (CIBIUG). The participants provided their written informed consent to participate in this study.

## Author Contributions

JM-S conceived and designed this study, oversaw the data collection, conducted the data analysis, and wrote the corresponding sections of the paper. LFS proposed the environmental dimensions, analyzed the data with MDS, categorized the places, wrote the corresponding sections, and concluded about the design guidelines. SR-C realized the structural equation modeling of the data. All the authors read, edited, and contributed to the writing of the final manuscript.

## Conflict of Interest

The authors declare that the research was conducted in the absence of any commercial or financial relationships that could be construed as a potential conflict of interest.

## Publisher's Note

All claims expressed in this article are solely those of the authors and do not necessarily represent those of their affiliated organizations, or those of the publisher, the editors and the reviewers. Any product that may be evaluated in this article, or claim that may be made by its manufacturer, is not guaranteed or endorsed by the publisher.
